# Author Correction: Comparative cellular analysis of motor cortex in human, marmoset and mouse

**DOI:** 10.1038/s41586-022-04562-y

**Published:** 2022-03-22

**Authors:** Trygve E. Bakken, Nikolas L. Jorstad, Qiwen Hu, Blue B. Lake, Wei Tian, Brian E. Kalmbach, Megan Crow, Rebecca D. Hodge, Fenna M. Krienen, Staci A. Sorensen, Jeroen Eggermont, Zizhen Yao, Brian D. Aevermann, Andrew I. Aldridge, Anna Bartlett, Darren Bertagnolli, Tamara Casper, Rosa G. Castanon, Kirsten Crichton, Tanya L. Daigle, Rachel Dalley, Nick Dee, Nikolai Dembrow, Dinh Diep, Song-Lin Ding, Weixiu Dong, Rongxin Fang, Stephan Fischer, Melissa Goldman, Jeff Goldy, Lucas T. Graybuck, Brian R. Herb, Xiaomeng Hou, Jayaram Kancherla, Matthew Kroll, Kanan Lathia, Baldur van Lew, Yang Eric Li, Christine S. Liu, Hanqing Liu, Jacinta D. Lucero, Anup Mahurkar, Delissa McMillen, Jeremy A. Miller, Marmar Moussa, Joseph R. Nery, Philip R. Nicovich, Sheng-Yong Niu, Joshua Orvis, Julia K. Osteen, Scott Owen, Carter R. Palmer, Thanh Pham, Nongluk Plongthongkum, Olivier Poirion, Nora M. Reed, Christine Rimorin, Angeline Rivkin, William J. Romanow, Adriana E. Sedeño-Cortés, Kimberly Siletti, Saroja Somasundaram, Josef Sulc, Michael Tieu, Amy Torkelson, Herman Tung, Xinxin Wang, Fangming Xie, Anna Marie Yanny, Renee Zhang, Seth A. Ament, M. Margarita Behrens, Hector Corrada Bravo, Jerold Chun, Alexander Dobin, Jesse Gillis, Ronna Hertzano, Patrick R. Hof, Thomas Höllt, Gregory D. Horwitz, C. Dirk Keene, Peter V. Kharchenko, Andrew L. Ko, Boudewijn P. Lelieveldt, Chongyuan Luo, Eran A. Mukamel, António Pinto-Duarte, Sebastian Preiss, Aviv Regev, Bing Ren, Richard H. Scheuermann, Kimberly Smith, William J. Spain, Owen R. White, Christof Koch, Michael Hawrylycz, Bosiljka Tasic, Evan Z. Macosko, Steven A. McCarroll, Jonathan T. Ting, Hongkui Zeng, Kun Zhang, Guoping Feng, Joseph R. Ecker, Sten Linnarsson, Ed S. Lein

**Affiliations:** 1 Allen Institute for Brain Science, Seattle, WA USA; 2 Department of Biomedical Informatics, Harvard Medical School, Boston, MA USA; 3 Department of Bioengineering, University of California, San Diego, La Jolla, CA USA; 4 The Salk Institute for Biological Studies, La Jolla, CA USA; 5 Department of Physiology and Biophysics, University of Washington, Seattle, WA USA; 6 Stanley Institute for Cognitive Genomics, Cold Spring Harbor Laboratory, Cold Spring Harbor, NY USA; 7 Department of Genetics, Harvard Medical School, Boston, MA USA; 8 LKEB, Department of Radiology, Leiden University Medical Center, Leiden, The Netherlands; 9 J. Craig Venter Institute, La Jolla, CA USA; 10 Genomic Analysis Laboratory, The Salk Institute for Biological Studies, La Jolla, CA USA; 11 Epilepsy Center of Excellence, Department of Veterans Affairs Medical Center, Seattle, WA USA; 12 Bioinformatics and Systems Biology Graduate Program, University of California, San Diego, La Jolla, CA USA; 13 Institute for Genomes Sciences, University of Maryland School of Medicine, Baltimore, MD USA; 14 Center for Epigenomics, Department of Cellular and Molecular Medicine, University of California, San Diego, La Jolla, CA USA; 15 Department of Computer Science, University of Maryland College Park, College Park, MD USA; 16 Ludwig Institute for Cancer Research, La Jolla, CA USA; 17 Sanford Burnham Prebys Medical Discovery Institute, La Jolla, CA USA; 18 Biomedical Sciences Program, School of Medicine, University of California, San Diego, La Jolla, CA USA; 19 University of Connecticut, Storrs, CT USA; 20 Computer Science and Engineering Program, University of California, San Diego, La Jolla, CA USA; 21 Department of Medical Biochemistry and Biophysics, Karolinska Institutet, Stockholm, Sweden; 22 McDonnell Genome Institute, Washington University School of Medicine, St Louis, MO USA; 23 Department of Physics, University of California, San Diego, La Jolla, CA USA; 24 Cold Spring Harbor Laboratory, Cold Spring Harbor, NY USA; 25 Departments of Otorhinolaryngology, Anatomy and Neurobiology, University of Maryland School of Medicine, Baltimore, MD USA; 26 Nash Family Department of Neuroscience and Friedman Brain Institute, Icahn School of Medicine at Mount Sinai, New York, NY USA; 27 Computer Graphics and Visualization Group, Delt University of Technology, Delft, The Netherlands; 28 Department of Physiology and Biophysics, Washington National Primate Research Center, University of Washington, Seattle, WA USA; 29 Department of Laboratory Medicine and Pathology, University of Washington, Seattle, WA USA; 30 Department of Neurological Surgery, University of Washington School of Medicine, Seattle, WA USA; 31 Regional Epilepsy Center, Harborview Medical Center, Seattle, WA USA; 32 Pattern Recognition and Bioinformatics group, Delft University of Technology, Delft, The Netherlands; 33 Department of Human Genetics, University of California, Los Angeles, Los Angeles, CA USA; 34 Department of Cognitive Science, University of California, San Diego, La Jolla, CA USA; 35 Broad Institute of MIT and Harvard, Cambridge, MA USA; 36 Department of Pathology, University of California, San Diego, CA USA; 37 Division of Vaccine Discovery, La Jolla Institute for Immunology, La Jolla, CA USA; 38 McGovern Institute for Brain Research, MIT, Cambridge, MA USA; 39 Department of Brain and Cognitive Sciences, MIT, Cambridge, MA USA; 40 Stanley Center for Psychiatric Research, Broad Institute of MIT and Harvard, Cambridge, MA USA; 41 Howard Hughes Medical Institute, The Salk Institute for Biological Studies, La Jolla, CA USA

**Keywords:** Molecular evolution, Cellular neuroscience, Genetics of the nervous system, Molecular neuroscience

Correction to: *Nature* 10.1038/s41586-021-03465-8 Published online 6 October 2021

In the version of this article initially published, the Acknowledgements section was incomplete and has now been amended to include the following: “NIH BRAIN Initiative awards U01 MH121282 to J.R.E and M.M.B, U19 MH114831 to J.R.E. and E.M.C., U19 MH114830 to H.Z., U01 MH114819 to G.F., 1U01MH114828 to K.Z. and J.C., RF1MH123220 to M.H. and R.H.S., and U19 MH114821. NIH awards R01DC019370 to R.H., R24MH114815 to R.H. and O.R.W., and R24 MH114788 to O.R.W. Nancy and Buster Alvord Endowment to C.D.K.” The changes have been made to the HTML and PDF versions of the article

