# Supplementary material for: Comparative cellular analysis of motor cortex in human, marmoset and mouse
Source: Nature. 2021 Oct 6;598(7879):111–9. doi: 10.1038/s41586-021-03465-8 (PMC8494640; doi:10.1038/s41586-021-03465-8)
Supplement: Supplementary file 1 — This file contains the legends for Supplementary Tables 1-23 (see separate zipped folder for the Table files). [file 41586_2021_3465_MOESM1_ESM.pdf]

nature portfolio

https://doi.org/10.1038/s41586-021-03465-8

Supplementary information

Comparative cellular analysis of motor cortex in human, marmoset and mouse

In the format provided by the authors and unedited

Supplementary Table legends

Supplementary Table 1. Provisional cell ontology (pCL) terms for human, mouse, and marmoset primary motor cortex cell types. Column headers are described as follows: pCL_id is a unique alphanumeric identifier assigned to each provisional cell type. CL_id is the Cell Ontology (CL) identifier for those parent cell type classes already represented in CL. pCL_name and Transcriptome data cluster are labels given according to each species naming convention that combines information about cortical layer enrichment and genes expressed in data cluster transcriptomes. TDC_id is a unique identifier assigned to the transcriptome data cluster. The part_of (uberon_id) and part_of (uberon_name) columns contain unique identifiers and names for tissue anatomic regions from which the experiment specimen was derived, in this case primary motor cortex. The is_a (CL or pCL_id) and is_a (CL or pCL_name) columns contain parent cell type or provisional cell type identifiers and names, respectively. Cluster_size indicates the number of single-nucleus or cell transcriptomes that were assigned membership to the transcriptome data cluster. Marker_gene_evidence indicates the number of marker genes that are necessary and sufficient to define the transcriptome cell type data cluster with maximal classification accuracy based on the NS-Forest v2.1 algorithm (see Supplementary Tables 4-6). F-measure_evidence is the f-beta score of classification accuracy from the NS-Forest v2.1 algorithm using the marker genes listed. The selectively_expresses column lists the minimum set of marker genes necessary and sufficient to define the transcriptome cell type data cluster. The definition brings together features to form a data driven ontological representation for each cell type cluster. The pCL annotations are available at https://github.com/mkeshk2018/Provisional_Cell_Ontology and https://bioportal.bioontology.org/ontologies/PCL.

Supplementary Table 2. Cluster annotations for human, marmoset, and mouse in separate worksheets. Cluster_label column identifies the RNA-seq cluster within each species. Cluster_size column denotes the number of nuclei that reside within each cluster (cluster_label). Class column identifies which cell class each cluster belongs to. Subclass column identifies which cell subclass each cluster belongs to. Cross-species cluster column indicates the cross-species consensus cluster taxonomy. DNAm_cluster_label column identifies the transcriptomic cluster (cluster_label) that is aligned to DNAm-determined clusters. ATAC_cluster label column identifies the transcriptomic cluster (cluster_label) that is aligned to ATAC-determined clusters.

Supplementary Table 3. Application of Allen Institute nomenclature schema to mouse, marmoset, macaque, human, and integrated M1 taxonomies. The “taxonomy_ids” tab lists ids and descriptions for the 11 taxonomies included and which tab those taxonomies are shown on. The “aligned_aliases” tab shows a list of aligned aliases for linking between taxonomies, as well as descriptions for these. The next five tabs show nomenclatures for each of the taxonomies and have the following column headers: “tree_order” is the order shown in the tree (if any); “cell_set_preferred_alias”, “cell_set_label”, and “cell_set_accession” are unique identifiers, as described in the Allen Institute nomenclature page (https://portal.brain-map.org/explore/classes/nomenclature), with “cell_set_preferred_alias” including the names used in this manuscript; “cell_set_aligned_alias” indicates which clusters correspond to the

“aligned_alias”es from the previous tab, if any; “cell_set_alias_integrated” shows linkages between single species transcriptomics taxonomies and the integrated taxonomy; “cell_set_labels_CCN##" columns indicate linkages between cell sets in the transcriptomics and other modalities within a single species; “cell_set_descriptor” shows the type of cell set (or level of ontology); “cell_set_alias_assignee” and “cell_set_alias_citation” indicate who defined the cell cet and in what publication; “cell_set_structure” and “cell_set_ontology_term” assign a specific brain structure to each cell set; and “taxonomy_id” links to the “taxonomy_id” tab. Finally, the “Cell class hierarchy” tab shows the ordered class, level2, and subclass hierarchy and associated colors used as cell sets in previous tabs. Code to generate cell type nomenclatures is available for download from https://github.com/AllenInstitute/nomenclature.

Supplementary Table 4. NS-Forest v2.1 was used to determine cell type cluster marker genes for all annotated levels of the human primary motor cortex cell type taxonomy defined by RNA-seq (Cv3). “clusterName” corresponds to the annotation label, either a cell type cluster name or a parent cell type class in the taxonomy. “markerCount” gives the optimal number of marker genes in the set that best discriminates the label. The “f-measure” column gives the f-beta score for classification using the set of markers. The next four columns “True Negative”, “False Positive”, “False Negative”, “True Positive” give the confusion matrix for the label given the set of markers. Finally, “Marker 1-5” lists the gene symbols corresponding to the optimal set of markers.

Supplementary Table 5. NS-Forest v2.1 was used to determine cell type cluster marker genes for all annotated levels of the mouse primary motor cortex cell type taxonomy defined by RNA-seq (Cv3). “clusterName” corresponds to the annotation label, either a cell type cluster name or a parent cell type class in the taxonomy. “markerCount” gives the optimal number of marker genes in the set that best discriminates the label. The “f-measure” column gives the f-beta score for classification using the set of markers. The next four columns “True Negative”, “False Positive”, “False Negative”, “True Positive” give the confusion matrix for the label given the set of markers. Finally, “Marker 1-5” lists the gene symbols corresponding to the optimal set of markers.

Supplementary Table 6. NS-Forest v2.1 was used to determine cell type cluster marker genes for all annotated levels of the marmoset primary motor cortex cell type taxonomy defined by RNA-seq (Cv3). “clusterName” corresponds to the annotation label, either a cell type cluster name or a parent cell type class in the taxonomy. “markerCount” gives the optimal number of marker genes in the set that best discriminates the label. The “f-measure” column gives the f-beta score for classification using the set of markers. The next four columns “True Negative”, “False Positive”, “False Negative”, “True Positive” give the confusion matrix for the label given the set of markers. Finally, “Marker 1-5” lists the gene symbols corresponding to the optimal set of markers.

Supplementary Table 7. DEGs determined by ROC test between each GABAergic neuron subclass and all other GABAergic nuclei within each species. Columns are labeled conservation, which indicates the species the gene was determined significant in; cluster,

denotes the target cluster the gene is a marker of; gene, indicating the gene that was identifies as DE; [species]_AUC, contains AUC scores > 0.7 if the gene was significant for a given subclass in a given species; [species]_power, contains the power of the ROC test; [species]_avg.1, contains the average log SCT-normalized expression of the target subclass; [species]_avg.2, contains the average log SCT-normalized expression of the non-target subclasses; [species]_prop.1, indicates the proportion of target subclass nuclei that express the gene; [species]_prop.2, indicates the proportion of non-target subclass nuclei that express the gene.

Supplementary Table 8. List of DEGs (from Supplementary Table 7) that is sorted according to the order the genes appear within the heatmap.

Supplementary Table 9. Supervised MetaNeighbor results, within- and across-species. Each row corresponds to a unique entry for a given gene set and a given cell class, either glutamatergic or GABAergic. The first five columns provide information about the gene sets, namely their provenance (SynGO or HGNC); numerical IDs; descriptive labels; manual classifications for plotting and interpretation; and finally the number of genes included in the analysis (after subsetting to genes with 1-1 orthologs across all three species). The sixth column indicates cell class. The remaining columns contain MetaNeighbor AUROCs for various analyses: within_species_meanROC (column 7) provides the mean of within-mouse (column 8), within-marmoset (column 9) and within-human (column 10) performance. For each species, tests were run with random 3-fold cross-validation, and the average across folds is reported. Columns 11 and 12 contain results from cross-species analyses, detailed in the methods. Results are sorted by their AUROC across primates (column 12).

Supplementary Table 10. DEGs determined by ROC test between each Glutamatergic neuron subclass and all other Glutamatergic nuclei within each species. Columns are labeled conservation, which indicates the species the gene was determined significant in; cluster, denotes the target cluster the gene is a marker of; gene, indicating the gene that was identified as DE; [species]_AUC, contains AUC scores > 0.7 if the gene was significant for a given subclass in a given species; [species]_power, contains the power of the ROC test; [species]_avg.1, contains the average log SCT-normalized expression of the target subclass; [species]_avg.2, contains the average log SCT-normalized expression of the non-target subclasses; [species]_prop.1, indicates the proportion of target subclass nuclei that express the gene; [species]_prop.2, indicates the proportion of non-target subclass nuclei that express the gene.

Supplementary Table 11. List of DEGs (from Supplementary Table 10) that is sorted according to the order the genes appear within the heatmap.

Supplementary Table 12. Average expression of isoforms in human and mouse subclasses and estimates of isoform genic proportions (P) based on the ratio of isoform to gene expression. Isoforms were included if they had at least moderate expression (TPM > 10) and P > 0.2 in either human or mouse and at least moderate gene expression (TPM > 10) in both species.

Supplementary Table 13. SNARE-seq2 metadata, cluster annotations and quality statistics. Tab 13a indicates SNARE-seq2 experiment level metadata (experiment name, library, patient, species, purification, age, sex) and mapping statistics for RNA (mean UMI detected, mean genes detected) and AC (mean fraction of reads in promoters or FRiP, mean uniquely mapped fragments grouped by 5000 base pair chromosomal bins, mean unique fragment counts per final peak locations, total number of final nuclei, total number of fragments, uniquely mapped fragments, properly paired fragments, unique (distinct) fragments). Tab 13b indicates the SNARE-seq2 local RNA clusters for human M1 generated using Pagoda2 (local cluster, annotated cluster name, broad cell type and abbreviation, k value used for Pagoda2 clustering, broad cell type markers, level 1 and level 2 classes and associated markers, unique cluster markers). Tabs 13c-d indicates SNARE-seq2 RNA and AC-level cluster annotations for human and marmoset M1, respectively, including annotated cluster name, cluster order, associated subclass and class, and the number of datasets making up the clusters. Tabs 13e-f lists all metadata outlined in tabs 13a-d for all SNARE-seq2 cell barcodes from human and marmoset M1 samples, respectively.

Supplementary Table 14. SNARE-seq2 differentially accessible regions (DARs) for human and marmoset M1. Tab 14a shows SNARE-seq2 DARs (q value < 0.01, log-fold change > 1) identified by AC-level clusters for human M1. Tab 14b shows corresponding AC-level cluster DARs that were linked to marker genes. Tab 14c shows subclass level DARs (q value < 0.001, log-fold change > 1) for human M1. Tab 14d shows subclass DARs (q value < 0.05, log-fold change > 1) for marmoset subclasses. Tab 14e shows sub-sampled subclass level DARs (q value < 0.005, log-fold change > 1, AUC > 0.5017) for human M1. Tab 14f shows sub-sampled subclass DARs (q value < 0.05, log-fold change > 1, AUC > 0.5064) for marmoset subclasses. Tab 14g shows a summarization of human and marmoset DARs detected by matched subclasses (sub-sampled), indicating actual number of DARs detected (tabs 14e and 14f). Tab 14h shows SNARE-seq2 DARs (q value < 0.05, log-fold change > 1) identified by RNA clusters for human M1. DAR tables indicate chromosomal location, p value (hypergeometric test), q value (Benjamini-Hochberg adjusted p value), log-fold change and associated cluster or subclass.

Supplementary Table 15. snmC-seq2 metadata. The table shows experiment level metadata, including species, sample name, gender, purification information, experiment nuclei numbers and pass-QC nuclei numbers.

Supplementary Table 16. Table of CHN and CGN marker genes for each snmC-seq2 cluster. Tabs denote species (human = hs, marmoset = cj, mouse = mm) CHN or CGN values. Columns indicate (from left to right) snmC-seq2 cluster label, gene symbols, average CHN or CGN methylation, logFC, p-values, and adjusted p-values. The markers were determined using a one-sided t-test with variance overestimated. The p-values are adjusted using a Benjamini-Hochberg procedure.

Supplementary Table 17. Differential TFBS activities for human and marmoset M1. ChromVAR differentially active TFBS activities between subclasses (human M1 - Tab 17a; marmoset M1 - 17b), AC-level clusters (human M1 - Tab 17c; marmoset M1 - Tab 17d), RNA clusters (human M1 - Tab 17e) and PVALB GABAergic cell types (Tab 17f) using a logistic regression test. Tables show p value, adjusted p values (Benjamini-Hochberg adjusted), average log fold change values (avg_logFC), the percent of nuclei showing accessibility for the TFBS in the target cluster (pct.1), the percent of non-target nuclei that show accessibility for the TFBS (pct.2).

Supplementary Table 18. Subclass TFBS enrichment results. TFBS enrichment analysis was done with AME [67] using JASPAR 2020 motifs. Within a species, hypo-methylated DMRs in each subclass were tested against hypo-methylated DMRs of all the other subclasses (background). DMRs and 250bp around regions were used in the analysis. A one-sided Fisher's exact test was used in AME [67]. This table includes p-values and effect sizes (log2(TP/FP)) of the analysis results.

Supplementary Table 19. Subclass TF enrichment at TF cluster level. The first column of the table lists the TF cluster categories according to JASPAR 2020 annotations [95]. The column names denote the subclass, species acronym (hs = human, cj = marmoset, mm = mouse), and modality (AC, DNAm, RNA). For AC, we report the average TFBS activity for each TF cluster (example column sst.hs.atac). For DNAm, the most significant log10 p-value for a TF in a given TF cluster is reported (example column sst.hs.dnam). We also report the largest effect size (log2FC) for a TF in a given TF cluster (example column sst.hs.dnam.effect.size). For RNA, we report the average log2(CP100k) expression for the highest expressed TF in each TF cluster (example column sst.hs.rna.log2cp100k). We also report the highest expressed TF gene symbol in each TF cluster (example column sst.hs.rna.max.gene).

Supplementary Table 20. DEGs determined by ROC test between chandelier cells and basket cells within each species. Columns are labeled as species, with true/false values indicating if a gene was enriched in chandelier cells for that species.

Supplementary Table 21. DEGs determined by ROC test between L5 ET subclass and L5 IT subclass within each species. Columns are labeled as species, with values of 1 indicating a gene was enriched in the L5 ET subclass for that species. A value of 0 indicates that the gene was not enriched in the L5 ET subclass for that species.

Supplementary Table 22. Genes with expression enrichment in L5 ET versus L5 IT that decreases with evolutionary distance from human (human > macaque > marmoset > mouse). Columns are labeled by species, and values indicate the log-fold change between L5 ET and L5 IT for that species. Genes were included if they had a minimum log-fold change greater than 0.5 in human.

Supplementary Table 23. List of marker genes identified from DNAm and/or Cv3 data that are used to integrate between the two modalities. The column of DNAm type shows whether CH or CG methylation is used for integration.
