# Supplementary material for: Comparative cellular analysis of motor cortex in human, marmoset and mouse
Source: Nature. 2021 Oct 6;598(7879):111–9. doi: 10.1038/s41586-021-03465-8 (PMC8494640; doi:10.1038/s41586-021-03465-8)
Supplement: Supplementary file 2 — Reporting Summary [file 41586_2021_3465_MOESM2_ESM.pdf]

nature research

Corresponding author(s): Ed S. Lein and Trygve E. Bakken

Last updated by author(s): Dec 7, 2020

# Reporting Summary

Nature Research wishes to improve the reproducibility of the work that we publish. This form provides structure for consistency and transparency in reporting. For further information on Nature Research policies, see Authors & Referees and the Editorial Policy Checklist.

## Statistics

For all statistical analyses, confirm that the following items are present in the figure legend, table legend, main text, or Methods section.

| n/a | Confirmed | |
|---|---|---|
| ☐ | ☒ | The exact sample size (*n*) for each experimental group/condition, given as a discrete number and unit of measurement |
| ☐ | ☒ | A statement on whether measurements were taken from distinct samples or whether the same sample was measured repeatedly |
| ☐ | ☒ | The statistical test(s) used AND whether they are one- or two-sided<br>*Only common tests should be described solely by name; describe more complex techniques in the Methods section.* |
| ☒ | ☐ | A description of all covariates tested |
| ☐ | ☒ | A description of any assumptions or corrections, such as tests of normality and adjustment for multiple comparisons |
| ☐ | ☒ | A full description of the statistical parameters including central tendency (e.g. means) or other basic estimates (e.g. regression coefficient) AND variation (e.g. standard deviation) or associated estimates of uncertainty (e.g. confidence intervals) |
| ☐ | ☒ | For null hypothesis testing, the test statistic (e.g. *F*, *t*, *r*) with confidence intervals, effect sizes, degrees of freedom and *P* value noted<br>*Give P values as exact values whenever suitable.* |
| ☒ | ☐ | For Bayesian analysis, information on the choice of priors and Markov chain Monte Carlo settings |
| ☒ | ☐ | For hierarchical and complex designs, identification of the appropriate level for tests and full reporting of outcomes |
| ☐ | ☒ | Estimates of effect sizes (e.g. Cohen's *d*, Pearson's *r*), indicating how they were calculated |

*Our web collection on statistics for biologists contains articles on many of the points above.*

## Software and code

Policy information about availability of computer code

| | |
|---|---|
| Data collection | BD Diva software v8.0, Nikon NIS-Elements Advanced Research imaging software v4.20, SoftMax Pro v6.5; VWorks v11.3.0.1195 and v13.1.0.1366; Hamilton Run Time Control v4.4.0.7740; Fragment Analyzer v1.2.0.11; Mantis Control Software v3.9.7.19; 10x Chromium v3 and Illumina MiSeq, HiSeq 2500, and Novaseq 6000 instrument control software. Physiology data acquisition software was either MIES (https://github.com/AllenInstitute/MIES/) or custom software written in Igor Pro. |
| Data analysis | Smart-seq v4 paired-end reads were clipped using ea-utils, then mapped using Spliced Transcripts Alignment to a Reference (STAR v2.7.3a). Reads were quantified using the R package, GenomicAlignments v1.18.0. For 10x Cv3 datasets, gene expression was quantified using 10x Cell Ranger v3 (https://support.10xgenomics.com/single-cell-gene-expression/software/downloads/latest). The clustering pipeline is implemented in the R package, scrattch.hicat v0.0.22 (https://github.com/AllenInstitute/scrattch.hicat). Custom R code written for clustering, and marker gene analysis and using open source R packages is available from https://github.com/AllenInstitute/BICCN_M1_Evo. This includes R packages fastICA v1.2-1, limma v3.38.3, MetaNeighbor v1.9.1 (https://github.com/gillislab/MetaNeighbor), scrattch.io v0.1.0, ggplot2 v3.3.2, Pagoda2 v0.1.0 (https://github.com/hms-dbmi/pagoda2), corrplot v0.84 (https://github.com/taiyun/corrplot), igraph v1.2.6, Seurat v3.1.1 (https://satijalab.org/seurat/), eulerr v6.0.0, UpSetR v1.4.0, RSEM v1.3.3. Python packages DoubletDetection v2.5 and NSforest v2.1 (https://github.com/JCVenterInstitute/NSForest). Cross-species tree merging algorithm available at https://github.com/huqiwen0313/speciesTree. DNA-methylation and RNA-seq integration used custom code at https://github.com/lhqing/cemba_data.<br><br>ATAC-seq data analysis using R packages SnapATAC v2 (https://github.com/r3fang/SnapATAC), DropletUtils v1.6.1, Cicero v1.2.0 (https://cole-trapnell-lab.github.io/cicero-release/), chromVAR v1.8.0 (https://greenleaflab.github.io/chromVAR), chromfunks v0.3.0 (https://github.com/yanwu2014/chromfunks), SWNE v0.5.7 (https://github.com/yanwu2014/swne), Signac v0.1.4 (https://satijalab.org), pROC v1.16.2, GenomicRanges v1.38.0, Gviz v1.30.3, and EdgeR v3.28.1. Python 3.70 packages Bowtie v1.1.0, samtools v1.9, Phenograph v1.5.2, Snaptools v1.4.7, MACS2 v2.1.2 (https://github.com/taoliu/MACS), and deepTools v3.4.2.<br><br>SnmC-seq2-seq data analysis using Python software scanpy v1.4.4, scikit-learn v0.21.3, imblearn v0.0, Scanorama v1.0, methylpy v1.4.0 (https://github.com/yupenghe/methylpy). |

Genome browser tracks were generated using the Integrative Genomics Viewer (IGV v2.7.0). Neuron morphology reconstruction used ZEN 2012 SP2 software and Vaa3D v3.475. FIJI distribution of ImageJ v1.52p, GraphPad Prism v7.04.

For manuscripts utilizing custom algorithms or software that are central to the research but not yet described in published literature, software must be made available to editors/reviewers. We strongly encourage code deposition in a community repository (e.g. GitHub). See the Nature Research guidelines for submitting code & software for further information.

## Data

Policy information about availability of data

All manuscripts must include a data availability statement. This statement should provide the following information, where applicable:

- Accession codes, unique identifiers, or web links for publicly available datasets
- A list of figures that have associated raw data
- A description of any restrictions on data availability

Raw sequence data are available for download from the Neuroscience Multi-omics Archive (https://nemoarchive.org/) under accession number 'dat-ek5dbmu' and the Brain Cell Data Center (https://biccn.org/data). Visualization and analysis tools are available at NeMO Analytics (Individual species: https://nemoanalytics.org//index.html?layout_id=ac9863bf; Integrated species: https://nemoanalytics.org//index.html?layout_id=34603c2b) and Cytosplore Viewer (https://viewer.cytosplore.org/). These tools allow users to compare cross-species datasets and consensus clusters via genome and cell browsers and calculate differential expression within and among species. Subclass level methylome tracks can be found at http://neomorph.salk.edu/aj2/pages/cross-species-M1/. A semantic representation of the cell types defined through these studies is available in the provisional Cell Ontology (https://bioportal.bioontology.org/ontologies/PCL; Supplementary Table 1).

The following publicly available datasets were used for analysis: Jaspar motifs database (JASPAR2020, all vertebrate, http://jaspar.genereg.net/matrix-clusters/), HUGO Gene Nomenclature Committee (HGNC) at the European Bioinformatics Institute (https://www.genenames.org; downloaded January 2020), Synaptic Gene Ontology (SynGO; downloaded February 2020), and orthologous genes across species from NCBI Homologene (downloaded November 2019). Macaque reconstructions were from source data available in Neuromorpho (chandelier cell NeuroMorpho.org ID: NMO_01873, basket cell NeuroMorpho.org ID: NMO_01851). Mouse ATAC-seq available from https://assets.nemoarchive.org/dat-7qjdj84. MTG human SMARTseq v4 data (https://portal.brain-map.org/atlases-and-data/rnaseq/human-mtg-smart-seq, https://assets.nemoarchive.org/dat-swzf4kc). ENCODE blacklist regions (http://mitra.stanford.edu/kundaje/akundaje/release/blacklists/hg38-human/hg38.blacklist.bed.gz)

# Field-specific reporting

Please select the one below that is the best fit for your research. If you are not sure, read the appropriate sections before making your selection.

☒ Life sciences ☐ Behavioural & social sciences ☐ Ecological, evolutionary & environmental sciences

For a reference copy of the document with all sections, see nature.com/documents/nr-reporting-summary-flat.pdf

# Life sciences study design

All studies must disclose on these points even when the disclosure is negative.

**Sample size**

Sample size was not pre-determined. For RNA-seq, snmC-seq2, SNARE-seq2, snATAC-seq, and mFISH, single nuclei were isolated from postmortem brains of human (n = 5), macaque (n = 3), marmoset (n = 3), and mouse (n = 12-24). For human and marmoset, this allowed us to collect nuclei from high quality specimens that met stringent quality control metrics while also confirming that transcriptomic and epigenomic clusters were consistent between donors and not driven by technical artifacts.

For mice, sample size (number of animals) was determined by the experimental requirements for collection of sufficient tissue for each assay. In no case were differences between individual animals or batches similar in magnitude to the reported cell type differences. The number of cells collected was determined by specific limitations of each data modality.

**Data exclusions**

Low-quality nuclei were included for analysis if they met the following pre-established quality control (QC) thresholds.

Human RNA-seq (SMART-seq v4):
> 30% cDNA longer than 400 base pairs
> 500,000 reads aligned to exonic or intronic sequence
> 40% of total reads aligned
> 50% unique reads
> 0.7 TA nucleotide ratio

Human and Macaque RNA-seq (10x v3):
> 500 (non-neuronal nuclei) or > 1000 (neuronal nuclei) genes detected
< 0.3 doublet score

Marmoset RNA-seq (10x v3):
Cell barcodes were filtered to distinguish true nuclei barcodes from empty beads and PCR artifacts by assessing proportions of ribosomal and mitochondrial reads, ratio of intronic/exonic reads (> 50% of intronic reads), library size (> 1000 UMIs) and sequencing efficiency (true cell barcodes have higher reads/UMI).

Mouse RNA-seq (SMART-seq v4 and 10x v3):
< 100,000 total reads, < 1,000 detected genes (CPM > 0), < 75% of reads aligned to genome, or CG dinucleotide odds ratio > 0.5. Cells were classified into broad classes of excitatory, inhibitory, and non-neuronal based on known markers, and cells with ambiguous identities were removed as doublets.

snmC-seq2:
1) mCCC rate < 0.03. mCCC rate reliably estimates the upper bound of bisulfite non-conversion rate 5; 2) overall mCG rate > 0.5; 3) overall mCH rate < 0.2; 4) total final reads > 500,000; and 5) bismark mapping rate > 0.5.

SNARE-seq2:
RNA quality filtering. Empty barcodes were removed using the emptyDrops() function of DropletUtils 80, mitochondrial transcripts were removed, doublets were identified using the DoubletDetection software 81 and removed. All samples were combined across experiments within species and cell barcodes having greater than 200 and less than 7500 genes detected were kept for downstream analyses. To further remove low quality datasets, a gene UMI ratio filter (gene.vs.molecule.cell.filter) was applied using Pagoda2 (https://github.com/hms-dbmi/pagoda2). AC quality filtering. Cell barcodes were included if they showed greater than 1000 read fragments and 500 UMI. Read fragments were then binned to 5000 bp windows of the genome and only cell barcodes showing the fraction of binned reads within promoters greater than 10% (15% for marmoset) and less than 80% were kept.

Patch-seq:
Patch-seq nuclei were mapped to each species glutamatergic reference cell types and were retained for analysis if they mapped with > 85% confidence to a cluster in the L5 ET subclass.

**Replication**

Flow cytometry data were reproducible across human tissue specimens from the 5 donors used in the study and across different nuclei isolations from individual tissue donors.

RNA-seq: Clustering reproducibility was measured by performing clustering analysis 100 times using a randomly-selected 80% of nuclei.

snmC-seq2: Leiden clustering resolution parameter was selected by three criteria: 1. The portion of outliers < 0.05 in the final consensus clustering version. 2. The ultimate prediction model accuracy > 0.95. 3. The average cell per cluster ≥ 30, which controls the cluster size to reach the minimum coverage required for further epigenome analysis such as DMR calls. All three criteria prevented the over-splitting of the clusters.

SNARE-seq2: Clustering of RNA data using the Pagoda2 package was highly similar to results from mapping to RNA-seq clusters using a centroid-based classifier.

For in situ hybridization and immunohistochemistry experiments, the number of times an experiment was repeated with similar results is listed in relevant figure legends. In general, experiments using human tissues were repeated on at least 2 independent donor tissues.

**Randomization**

All species specimens were controls and were therefore allocated into the same experimental group. Randomization was not used.

**Blinding**

Human specimens were de-identified and assigned a unique numerical code. Researchers responsible for data generation and analysis were not blinded and had access to basic information about donors (age, sex, ethnicity), as well as the unique numerical code assigned to each donor.

For experiments other than those involving human specimens, similar donor information was available to researchers involved in data generation and analysis.

Blinding was not relevant to these experiments because species information was necessary for sequence alignment to correct reference genomes, integration pipelines, and was a primary analytical endpoint. Similarly, donor information was necessary for study design and cluster curation steps during analysis.

# Reporting for specific materials, systems and methods

We require information from authors about some types of materials, experimental systems and methods used in many studies. Here, indicate whether each material, system or method listed is relevant to your study. If you are not sure if a list item applies to your research, read the appropriate section before selecting a response.

## Materials & experimental systems

| n/a | Involved in the study |
|---|---|
| ☐ | ☒ Antibodies |
| ☐ | ☒ Eukaryotic cell lines |
| ☒ | ☐ Palaeontology |
| ☐ | ☒ Animals and other organisms |
| ☐ | ☒ Human research participants |
| ☒ | ☐ Clinical data |

## Methods

| n/a | Involved in the study |
|---|---|
| ☒ | ☐ ChIP-seq |
| ☐ | ☒ Flow cytometry |
| ☒ | ☐ MRI-based neuroimaging |

# Antibodies

| | |
|---|---|
| Antibodies used | 1. Mouse anti-NeuN-PE conjugated, EMD Millipore, Milli-Mark, clone A60, #FCMAB317PE, 1:500<br>2. Mouse anti-GFAP, EMD Millipore, #MAB360, clone GA5, 1:500<br>3. Mouse anti-neurofilament-H, nonphosphorylated (NF-H, clone SMI 32), Biolegend, #801701, 1:250<br>4. Goat anti-mouse IgG(H+L) Alexa Fluor 568 conjugate, ThermoFisher Scientific, #A-11004, 1:500<br>5. Goat anti-mouse IgG(H+L) Alexa Fluor 594 conjugate, ThermoFisher Scientific, #A-11005, 1:500<br>6. Goat anti-mouse IgG(H+L) Alexa Fluor 647 conjugate, ThermoFisher Scientific, #A-21235, 1:500<br>7. Mouse IgG1,k PE Isotype control, clone MOPC-21,#555749, BD Pharmingen, 1:250 |
| Validation | 1. mouse anti-NeuN-PE conjugated EMD Millipore, Milli-Mark, clone A60, #FCMAB317PE: From the manufacturer's website: This Milli-Mark Anti-NeuN-PE Antibody, clone A60 is validated for use in flow cytometry for the detection of NeuN. Quality is evaluated by flow cytometry using U251 cells. The immunogen is purified cell nuclei from mouse brain. Species reactivity – human.<br><br>2. Mouse anti-GFAP, EMD Millipore, #MAB360, clone GA5, 1:500: routinely evaluated by Western Blot on Mouse brain lysates.<br><br>3. Mouse anti-neurofilament-H, nonphosphorylated (NF-H, clone SMI 32), Biolegend, 1:250: From the manufacturer's website: Each lot of this antibody is quality control tested by formalin-fixed paraffin-embedded immunohistochemical staining. Species reactivity - Human, Mouse, Rat, Other mammalian. This antibody reacts with a nonphosphorylated epitope in neurofilament H of most mammalian species. The manufacturer provides IHC, IF, and western blot validation data for the antibody on their website. For immunohistochemistry, a concentrationcrange of 1.0 - 5.0 µg/ml is suggested.<br><br>4. Goat anti-mouse IgG(H+L) Alexa Fluor 568 conjugate, ThermoFisher Scientific, #A-11004, 1:500: each lot of antibody is quality control tested using immunocytochemistry.<br><br>5. Goat anti-mouse IgG(H+L) Alexa Fluor 647 conjugate, ThermoFisher Scientific, #A-21235, 1:500: each lot of antibody is quality control tested using immunocytochemistry.<br><br>6. Mouse IgG1,k PE Isotype control, clone MOPC-21,#555749, BD Pharmingen, 1:250: From the manufacturer's website: The MOPC-21 immunoglobulin is a mouse myeloma protein. The MOPC-21 immunoglobulin was selected as an isotype control following screening for low background on a variety of mouse and human tissues. The monoclonal antibody was purified from tissue culture supernatant or ascites by affinity chromatography. The antibody was conjugated with R-PE under optimum conditions, and unconjugated antibody and free PE were removed. The manufacturer states that the antibody is routinely tested by flow cytometry. |

# Eukaryotic cell lines

Policy information about cell lines

| | |
|---|---|
| Cell line source(s) | HEK 293T/17 cell line was sourced from ATCC. Product page: https://www.atcc.org/products/all/CRL-11268.aspx# |
| Authentication | None of the cell lines used were authenticated. |
| Mycoplasma contamination | The cell lines were not tested for mycoplasma contamination. |
| Commonly misidentified lines<br>(See ICLAC register) | *Name any commonly misidentified cell lines used in the study and provide a rationale for their use.* |

# Animals and other organisms

Policy information about studies involving animals; ARRIVE guidelines recommended for reporting animal research

| | |
|---|---|
| Laboratory animals | Pig-tailed macaque (Macaca nemestrina): adult 12.3 year male, 3.9 year female, 17.2 year male<br>Marmoset (Callithrix jacchus): adult (1.9-3.1 years), male and female<br>Mouse (Mus musculus): adult (P56 +/- 3 days) wildtype C57Bl/6J, male and female<br><br>For patch-seq, mouse (Mus musculus) M1 tissue was obtained from 4-12 week old male and female mice from the following transgenic lines: Thy1h-eyfp (B6.Cg-Tg(Thy1-YFP)-HJrs/J, RRID:IMSR_JAX:003782), Etv1-egfp Tg(Etv1-EGFP)BZ192Gsat/Mmucd, RRID:MMRRC_011152-UCD, (etv1) mice maintained with the outbred Charles River Swiss Webster background (Crl:CFW(SW, RRID:IMSR_CRL:024), and C57BL/6-Tg(Pvalb-tdTomato)15Gfng/J, RRID:IMSR_JAX:027395.<br><br>Mice were provided food and water ad libitum and were maintained on a regular 12-h day/night cycle at no more than five adult animals per cage. |
| Wild animals | No wild animals were used in this study. |
| Field-collected samples | No field-collected samples were used in this study. |
| Ethics oversight | Mouse experiments were conducted in accordance with the US National Institutes of Health Guide for the Care and Use of |

Ethics oversight

Laboratory Animals under protocol numbers 0120-09-16, 1115-111-18, or 18-00006 and were approved by the Institutional Animal Care and Use Committee at University of Washington, Allen Institute for Brain Science, Salk Institute, or Massachusetts Institute of Technology. Marmoset experiments were approved by and in accordance with Massachusetts Institute of Technology IACUC protocol number 051705020. Macaque tissue used in this research was obtained from the University of Washington National Primate Resource Center, under a protocol approved by the University of Washington Institutional Animal Care and Use Committee.

Note that full information on the approval of the study protocol must also be provided in the manuscript.

## Human research participants

Policy information about studies involving human research participants

Population characteristics

Postmortem tissue donors used in the study:

Specimen ID Age Sex Race Cause of Death PMI (hr) Tissue RIN Hemisphere Sampled Data Type
H200.1023 43 F Iranian descent Mitral valve prolapse 18.5 7.4 ± 0.7 L SSv4
H200.1025 50 M Caucasian Cardiovascular 24.5 7.6 ± 1.0 L SSv4
H200.1030 54 M Caucasian Cardiovascular 25 7.7 ± 0.8 L SSv4
H18.30.001 60 F Unknown Car accident 18 7.9 ± 2.5 R SSv4, Cv3, SNARE-seq2, sn-methlyome
H18.30.002 50 M Unknown Cardiovascular 10 8.2 ± 0.4 R SSv4, Cv3, SNARE-seq2, snmC-seq2

RIN, RNA integrity number. Data type: SMART-Seqv4 (SSv4), 10x Genomics Chromium Single Cell 3' Kit v3 (Cv3), Single-Nucleus Chromatin Accessibility and mRNA Expression sequencing (SNARE-seq2), Single nucleus methyl cytosine sequencing (snmC-seq2).

Neurosurgical tissue donor used in the study:
Donor was 61 F Unknown Glioblastoma grade IV, Patch-seq

Recruitment

Postmortem tissue specimens from males and females between 18 – 68 years of age with no known history of neuropsychiatric or neurological conditions ('control' cases) were considered for inclusion in this study of cell transcriptional profiles. Key conditions for exclusion were:

- Known brain injury, cancer or disease
- Known neuropsychiatric or neuropathological history
- Epilepsy or other seizure history
- Drug/alcohol dependency
- > 1 hour on ventilator
- Positive for infectious disease
- Prion disease
- Chronic renal failure
- Death from homicide or suicide
- Sleep apnea
- Time since death (postmortem interval, PMI) > 25 hours

Neurosurgical specimens: Tissue procurement from neurosurgical donor was performed outside of the supervision of the Allen Institute at a local hospital, and tissue was provided to the Allen Institute under the authority of the IRB of the participating hospital. A hospital-appointed case coordinator obtained informed consent from donor prior to surgery.

Ethics oversight

Postmortem adult human brain tissue was collected after obtaining permission from decedent next-of-kin. Postmortem tissue collection was performed in accordance with the provisions of the United States Uniform Anatomical Gift Act of 2006 described in the California Health and Safety Code section 7150 (effective 1/1/2008) and other applicable state and federal laws and regulations. The Western Institutional Review Board reviewed tissue collection processes and determined that they did not constitute human subjects research requiring institutional review board (IRB) review.

Tissue procurement from neurosurgical donor was performed outside of the supervision of the Allen Institute at a local hospital, and tissue was provided to the Allen Institute under the authority of the institutional review board of the participating hospital. A hospital-appointed case coordinator obtained informed consent from donor before surgery. Tissue specimens were de-identified before receipt by Allen Institute personnel. The specimens collected for this study were apparently non-pathological tissues removed during the normal course of surgery to access underlying pathological tissues. Tissue specimens collected were determined to be non-essential for diagnostic purposes by medical staff and would have otherwise been discarded.

Note that full information on the approval of the study protocol must also be provided in the manuscript.

# Flow Cytometry

## Plots

Confirm that:

☒ The axis labels state the marker and fluorochrome used (e.g. CD4-FITC).

☒ The axis scales are clearly visible. Include numbers along axes only for bottom left plot of group (a 'group' is an analysis of identical markers).

☒ All plots are contour plots with outliers or pseudocolor plots.

☒ A numerical value for number of cells or percentage (with statistics) is provided.

## Methodology

| | |
|---|---|
| Sample preparation | Microdissected tissue pieces were placed in into nuclei isolation medium containing 10mM Tris pH 8.0 (Ambion) , 250mM sucrose, 25mM KCl (Ambion), 5mM MgCl2 (Ambion) 0.1% Triton-X 100 (Sigma Aldrich), 1% RNasin Plus, 1X protease inhibitor (Promega), and 0.1mM DTT in 1ml dounce homogenizer (Wheaton). Tissue was homogenized using 10 strokes of the loose dounce pestle followed by 10 strokes of the tight pestle and the resulting homogenate was passed through 30µm cell strainer (Miltenyi Biotech) and centrifuged at 900xg for 10 min to pellet nuclei. Nuclei were resuspended in buffer containing 1X PBS (Ambion), 0.8% nuclease-free BSA (Omni-Pur, EMD Millipore), and 0.5% RNasin Plus. Mouse anti-NeuN conjugated to PE (EMD Millipore) was added to preparations at a dilution of 1:500 and samples were incubated for 30 min at 4°C. Control samples were incubated with mouse IgG1,k-PE Isotype control (BD Pharmingen). Samples were then centrifuged for 5 min at 400xg to pellet nuclei and pellets were resuspended in 1X PBS, 0.8% BSA, and 0.5% RNasin Plus. DAPI (4′, 6-diamidino-2-phenylindole, ThermoFisher Scientific) was applied to nuclei samples at a concentration of 0.1µg/ml. |
| Instrument | Single nucleus sorting was carried out on either a BD FACSAria II SORP or BD FACSAria Fusion instrument (BD Biosciences) |
| Software | BD Diva Software V8.0 |
| Cell population abundance | We intentionally sorted ~10% NeuN-negative (non-neuronal) and ~90% NeuN-positive (neuronal) nuclei to enrich for neurons. |
| Gating strategy | Nuclei were first gated based on size (forward scatter area, FSC-A) and granularity (side scatter area, SSC-A). B, Nuclei were then gated on DAPI fluorescence, followed by gates to exclude doublets and aggregates (FSC-single cells, SSC-single cells). E, Lastly, nuclei were gated based on NeuN PE ignal (NeuN-PE-A) to differentiate neuronal (NeuN+) and non-neuronal (NeuN-) nuclei. |

☒ Tick this box to confirm that a figure exemplifying the gating strategy is provided in the Supplementary Information.
